# Supplementary material for: Effects of Arbuscular Mycorrhizal Fungi on the Growth and Root Cell Ultrastructure of Eucalyptus grandis under Cadmium Stress
Source: J Fungi (Basel). 2023 Jan 19;9(2):140. doi: 10.3390/jof9020140 (PMC9964804; doi:10.3390/jof9020140)
Supplement: Supplementary file 1 [file jof-09-00140-s001.zip › jof-2143806-supplementary.pdf]

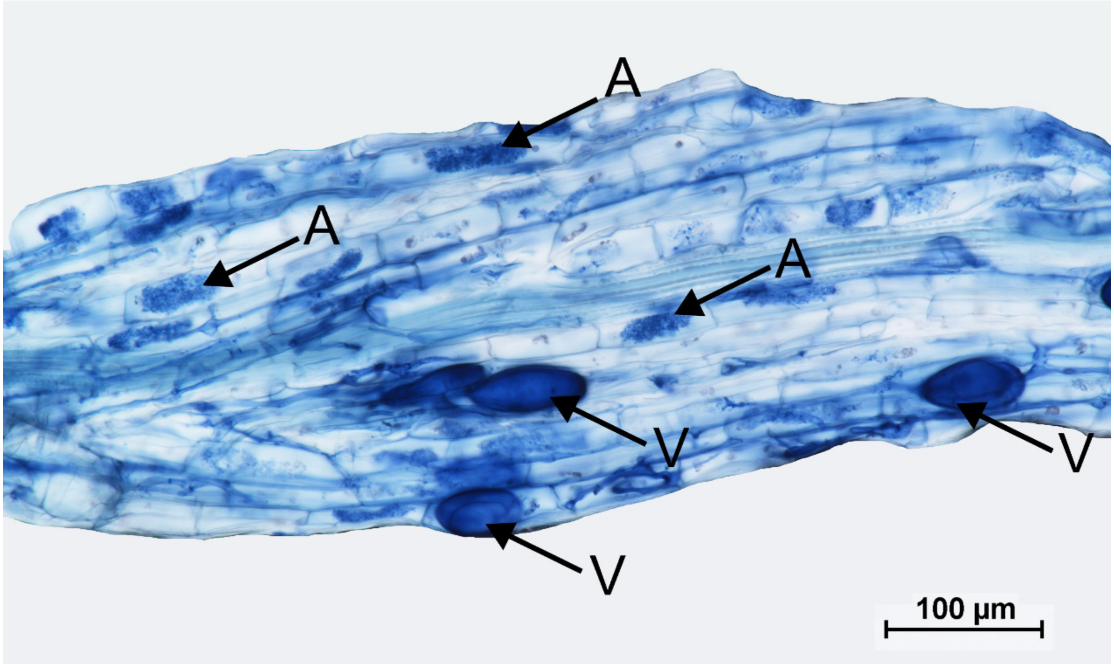

Figure S1. AMF colonization under non-stress condition (A: arbuscule; V: vesicle).

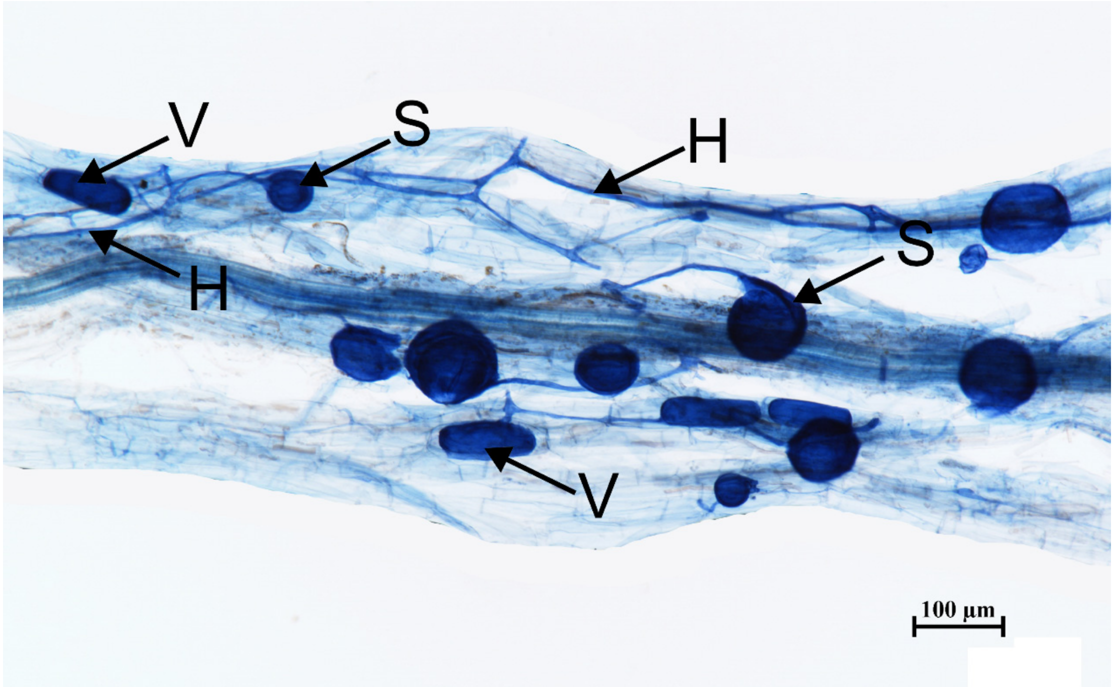

Figure S2. AMF colonization under 500 μM Cd concentration condition (V: vesicle; H: hypha; S: spore).

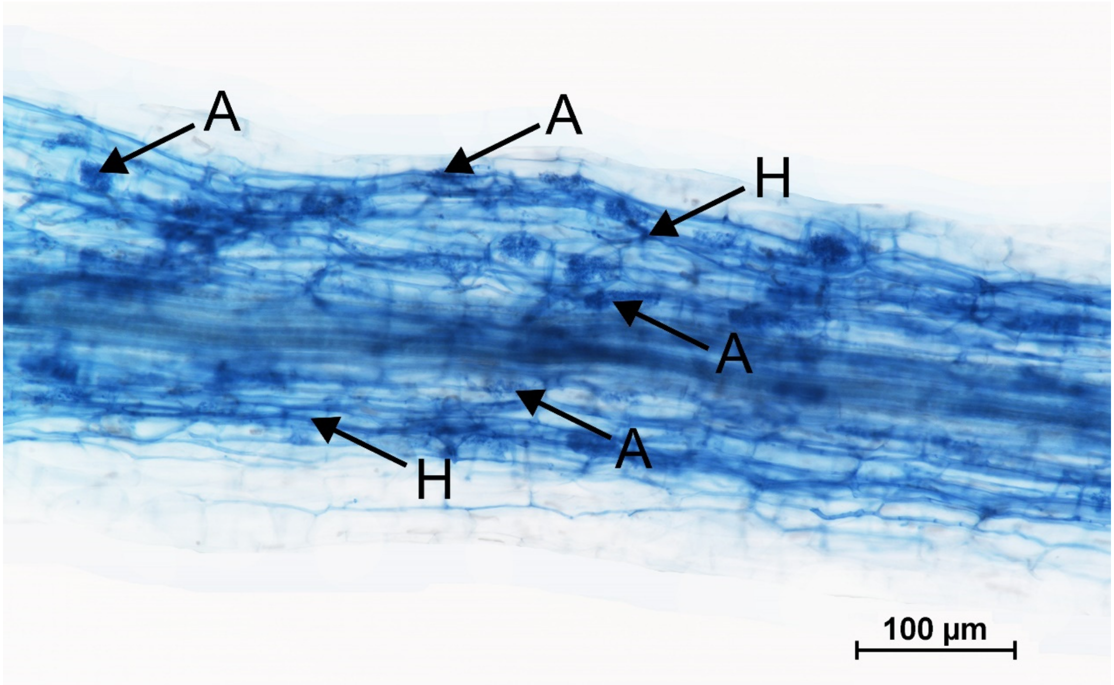

Figure S3. AMF colonization under 500 μM Cd concentration condition (A: arbuscule; V: vesicle; H: hypha).

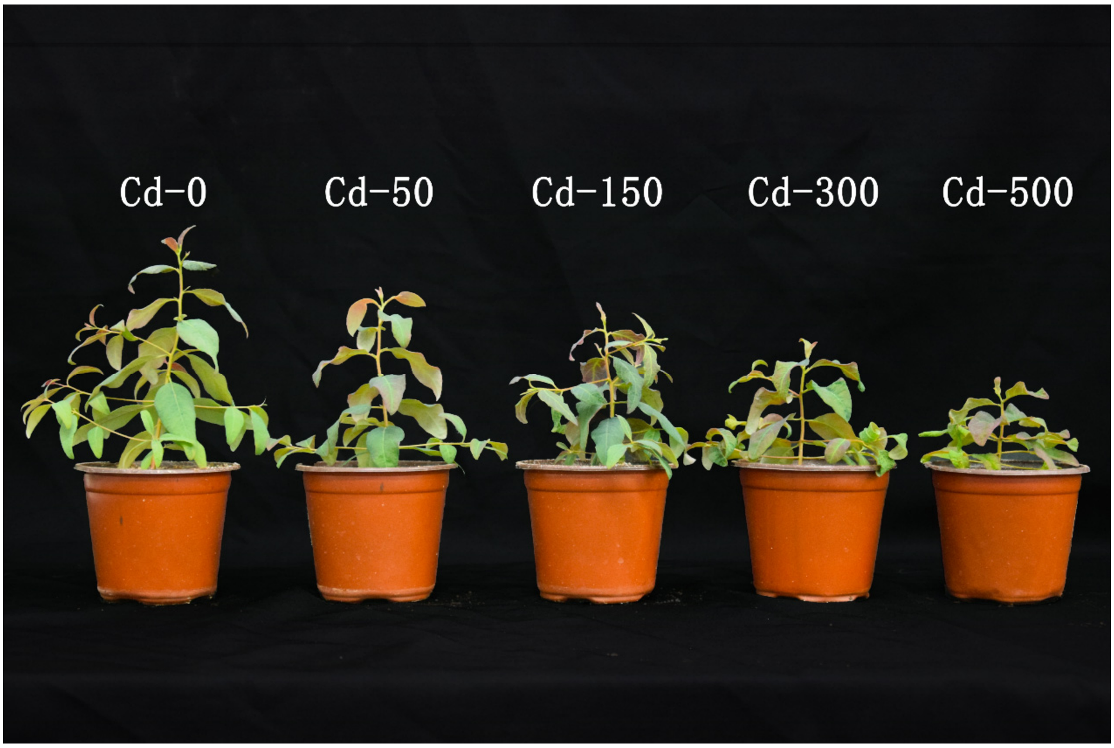

Figure S4. Effects of different Cd concentrations on the growth of *Eucalyptus grandis.*

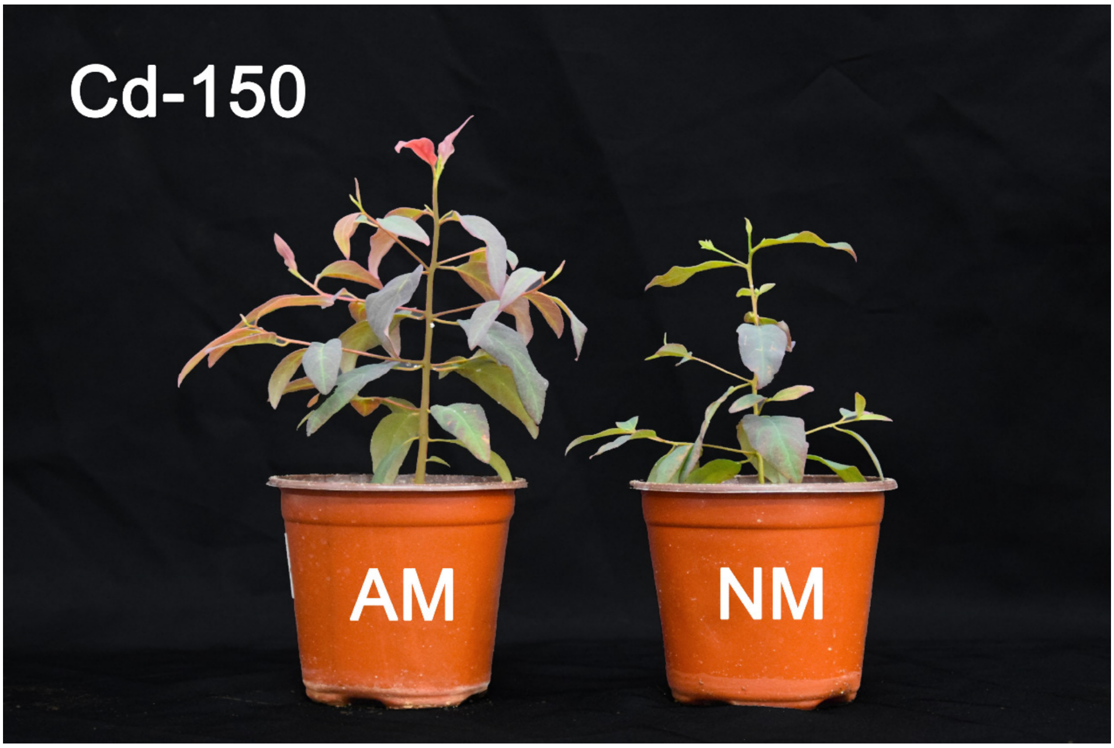

Figure S5. Effects of AMF colonization on the growth of *Eucalyptus grandis* under 150 µM Cd concentration condition.
